# Blood SIRT1 Shows a Coherent Association with Leptin and Adiponectin in Relation to the Degree and Distribution of Adiposity: A Study in Obesity, Normal Weight and Anorexia Nervosa

**DOI:** 10.3390/nu12113506

**Published:** 2020-11-14

**Authors:** Stefania Mariani, Maria Rosaria Di Giorgio, Erica Rossi, Rossella Tozzi, Savina Contini, Lisa Bauleo, Fiammetta Cipriani, Raffaella Toscano, Sabrina Basciani, Giuseppe Barbaro, Mikiko Watanabe, Agostino Valenti, Armando Cotugno, Carla Ancona, Carla Lubrano, Lucio Gnessi

**Affiliations:** 1 Department of Experimental Medicine, Section of Medical Physiopathology, Food Science and Endocrinology, “Sapienza” University of Rome, Rome, Italy; maridigiorgio@tiscali.it (M.R.D.G.); ericaro1993@gmail.com (E.R.); savina.contini@uniroma1.it (S.C.); fiammetta.cipriani@gmail.com (F.C.); raffaella.toscano@uniroma1.it (R.T.); sabrinabasciani@yahoo.it (S.B.); giuseppe.barbaro@uniroma1.it (G.B.); mikiko.watanabe@uniroma1.it (M.W.); carla.lubrano@uniroma1.it (C.L.); lucio.gnessi@uniroma1.it (L.G.); 2 Department of Molecular Medicine, “Sapienza” University of Rome, 00161 Rome, Italy; rossella.tozzi@uniroma1.it; 3 Department of Epidemiology, Lazio Regional Health Service, 00147 Rome, Italy; l.bauleo@deplazio.it (L.B.); c.ancona@deplazio.it (C.A.); 4 Internal Medicine, Santo Spirito in Sassia Hospital, 00193 Rome, Italy; agostino.valenti@aslroma1.it; 5 Department of Mental Health, UOSD eating behavior disorders, Padiglione I, Comprensorio S. Maria della Pietà, 00135 Rome, Italy; armando.cotugno@aslroma1.it

**Keywords:** adipose tissue, sirtuins, plasma SIRT1, adiponectin, leptin, anorexia nervosa, obesity

## Abstract

Sirtuin 1 (SIRT1) is a sensor of cell energy availability, and with leptin and adiponectin, it regulates metabolic homeostasis. Widely studied in tissues, SIRT1 is under evaluation as a plasmatic marker. We aimed at assessing whether circulating SIRT1 behaves consistently with leptin and adiponectin in conditions of deficiency, excess or normal fat content. Eighty subjects were evaluated: 27 with anorexia nervosa (AN), 26 normal-weight and 27 with obesity. Bloodstream SIRT1, leptin and adiponectin (ELISA), total and trunk fat mass (FM) %, abdominal visceral adipose tissue, liver steatosis and epicardial fat thickness (EFT) were assessed. For each fat store, the coefficient of determination (R^2^) was used to evaluate the prediction capability of SIRT1, leptin and adiponectin. Plasma SIRT1 and adiponectin coherently decreased with the increase of FM, while the opposite occurred with leptin. Mean levels of each analyte were different between groups (*p* < 0.005). A significant association between plasma variables and FM depots was observed. SIRT1 showed a good predictive strength for FM, particularly in the obesity group, where the best R^2^ was recorded for EFT (R^2^ = 0.7). Blood SIRT1, adiponectin and leptin behave coherently with FM and there is synchrony between them. The association of SIRT1 with FM is substantially superimposable to that of adiponectin and leptin. Given its homeostatic roles, SIRT1 may deserve to be considered as a plasma clinical/biochemical parameter of adiposity and metabolic health.

## 1. Introduction

The abnormal amount of fat mass (FM) implies dysfunction of adipose tissue. Fat excess as well as fat deficiency may result in significant changes in the levels of bloodstream adipokines and adipose tissue energy sensors. Adipokines, a large group of substances secreted by the white adipose tissue, regulate a range of homeostatic processes including energy metabolism. In patients suffering from obesity, the alteration of adipokine expression underlies the increased risk for inflammation and metabolic derangement, sustaining the development of cardiovascular, neurodegenerative and diabetic complications [1,2]. However, even in subjects with anorexia nervosa (AN), which show reduced fat depots and fat distribution mostly in the visceral districts with preferential subcutaneous fat loss from the extremities [3], changes in adipokine expression can lead to adipose tissue inflammation or ectopic fat distribution.

Leptin and adiponectin have long been studied in human and animal models [1,4]. Leptin, a hormone dependent on fat amount, acts as afferent signal in a hypothalamic negative feedback loop that maintains homeostatic control of FM [5]. In conditions of fat excess, leptin plays an anorectic action which, from an evolutionary point of view, protects the subject from gaining too much weight. Adiponectin, the circulating levels of which negatively correlate with obesity, increases the adipose tissue metabolic flexibility [6] and has antiatherogenic, anti-inflammatory, immunomodulatory, vasoprotective and insulin-sensitizing properties [7]. Nutrient restriction increases adiponectin in subcutaneous adipose tissue [8], and the excess of visceral fat implies adiponectin decrease, followed by a pro-inflammatory state associated with diseases [2]. 

In the adipose tissue, cellular energy sensors—deacetylases named sirtuins (SIRTs)—cooperate with adipokines. SIRT1, the most studied family member, has a regulatory role mainly in metabolism and aging, extending health span through effects against metabolic and degenerative diseases [9]. SIRT1 modulates gluconeogenesis in the liver, pancreatic ß cell insulin secretion [10], adipocyte differentiation [11] and heart protection [12]. In the case of overnutrition, tissue and plasma levels of SIRT1 are reduced, predisposing to metabolic derangement and its sequelae [13,14]. Indeed, SIRT1 inversely correlates with FM, and its reduction is associated with liver steatosis and increased epicardial fat thickness (EFT) in obese patients [15,16,17]. On the contrary, caloric restriction (CR) powerfully stimulates the activation of SIRT1 and improves general health [18,19,20]. Accordingly, SIRT1 transgenic mice show phenotypes resembling CR [21]. 

The prevalence of obesity is rising [22,23], and with this, many of its complications are increasing, some of which are well acknowledged, such as type 2 diabetes, obstructive sleep apnea syndrome, non-alcoholic fatty liver disease and cardiovascular disease [24,25,26,27], and others are emerging and currently being investigated [28,29]. Several strategies have been proposed for the treatment of weight excess and its detrimental consequences, ranging from dietary regimens [30,31,32] to pharmacological treatments [33], physical exercise [34] and psychological approaches [35]. Although all lead to improvement in many cases, none are always effective. 

The alterations in peripheral energy status sensing involving leptin, adiponectin and SIRT1 may lead to the alteration of energy balance and adiposity. Better understanding the underlying mechanisms, together with identifying feasible markers of unhealthy adiposity, could aid in the development of new tools to modulate body weight, and it is therefore crucial to investigate these aspects.

In this study, we analyzed SIRT1, leptin and adiponectin plasma concentrations in AN, normal weight (NW) and obesity in relation to different FM depots, including visceral fat, to evaluate whether circulating SIRT1 is coherently associated with leptin and adiponectin in each of the assessed conditions.

## 2. Materials and Methods 

### 2.1. Study Design and Population

Participants to this cross-sectional study were recruited at the Center of High Specialization for the Treatment of Obesity (CASCO) of the Department of Experimental Medicine, “Sapienza” University of Rome, the Santo Spirito Hospital of Rome, and the Department of Mental Health, UOSD eating behavior disorders, S. Maria della Pietà, Rome, Italy, in the period between February 2017 and December 2019. During the two years of recruitment, 51 patients suffering from AN, 146 NW subjects and 380 patients affected by obesity were evaluated. The diagnosis of AN was made according to the criteria reported in the DSM-5.

We included in the study patients with a body mass index (BMI) <18.5 kg/m^2^ for AN, between 18.5 and 24.9 kg/m^2^ for NW and ≥30 kg/m^2^ for obesity; absence of pregnancy; and no acute disease that occurred in the three months prior to the enrollment visit. Patients with heart and respiratory diseases, type 1 diabetes mellitus and uncontrolled type 2 diabetes, systemic corticosteroid therapy, any type of therapy that could potentially alter body weight and composition, full-blown hypothyroidism, cirrhosis and other chronic liver diseases, including those from hepatitis B or C and excessive alcohol consumption (≥140 g/week for men and ≥70 g/week for women), were excluded. 

After screening, 80 patients were enrolled: 27 affected by AN (weight stable for at least 1 month before the study), 26 NW and 27 affected by obesity. 

**Figure 1:**
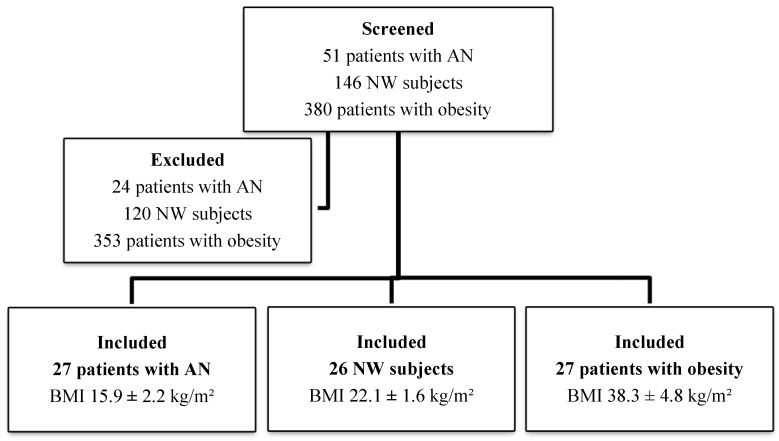
Patient selection flowchart. AN: anorexia nervosa; NW: normal weight; BMI: body mass index.

Figure 1 shows the patient enrollment flowchart and the study sample stratification according to BMI. Physical examination, anthropometric measurements and SIRT1, adiponectin and leptin assays were performed for all patients entering the study. Percentage of total body and trunk fat and abdominal visceral adipose tissue (VAT) were assessed by dual-energy X-ray absorptiometry (DEXA); liver steatosis and epicardial fat thickness (EFT) were measured by ultrasound. 

The study protocol was approved by the local Ethics Committee in agreement with the Helsinki Declaration (ref. CE 5475). All the subjects gave written informed consent before entering the study.

### 2.2. Plasma SIRT1, Adiponectin and Leptin Assay

SIRT1, adiponectin and leptin plasma concentrations were measured after an overnight fast. The SIRT1 levels were determined according to the manufacturer’s instructions using quantitative ELISAs (MyBioSource, Cod. GDMBS705558) as previously described [13]. Adiponectin was measured by Human Total Adiponectin/Acrp30 Quantikine ELISA kit (DRP300, R&D Systems, Minneapolis, MN, USA). The inter- and intra-assay coefficients of variation were 6.9 and 4.7%, respectively, the sensitivity was 0.891 ng/mL and the cross-reactivity <0.5%. 

Quantitative determination of human leptin was carried out by a high-sensitivity ELISA kit (MyBioSource, Inc., San Diego, CA, USA, Cod. MBS9425103). The sensitivity was <0.266 ng/mL, and no significant cross-reactivity or interference between leptin and analogues was described. 

### 2.3. Body Composition Assessment 

#### 2.3.1. Dual-energy X-ray absorptiometry analysis

DEXA scans were performed by one single experienced technician according to the manufacturer’s instructions (Hologic Inc., Bedford, MA, USA, QDR 4500 W). Percentage body fat composition was measured as previously described in the whole body (%) and, with the use of specific anatomic landmarks determined by a standard software (Hologic Inc., Bedford, MA, USA, S/N47168 VER. 11.2), in the trunk [13,36]. The abdominal visceral adipose tissue (VAT) was measured separately (g). Limits of the abdominal VAT area were defined as follows: upper cut line by the lower edge of the rib cage; pelvic cut line just above the iliac crest; lateral lines positioned at the inner edge of the abdominal muscle wall, bilaterally. The coefficient of variation for FM was <1.5%.

#### 2.3.2. Determination of Liver Adiposity

Liver fat content was determined with an Esaote Medica device equipped with a convex 3.5 MHz probe (Esaote MyLab40, Esaote Europe B.V., Maastricht, The Netherlands) as previously described [13]. The severity of liver adiposity was based according to the brightness of the liver estimated as a numerical value (0 = absent lipid accumulation; 1 = mild lipid accumulation; 2 = moderate/severe lipid accumulation).

#### 2.3.3. Echocardiographic Epicardial Fat Thickness Measurements

EFT was determined with a high-resolution M-B-mode transthoracic echocardiography using a 2.5-MHz probe (Esaote MyLab40, Esaote Europe B.V., Maastricht, The Netherlands) as previously described [13].

### 2.4. Data Management and Statistical Analysis

The sample was grouped by AN (BMI <18.5 kg/m^2^), NW (BMI 18.5–24.9 kg/m^2^) and obesity (BMI ≥ 30 kg/m^2^), and the analysis stratified accordingly. Differences between groups were analyzed using ANOVA test. Mean and standard deviation (SD) were calculated for SIRT1, adiponectin, leptin, weight, BMI, total FM %, trunk FM %, abdominal VAT (g) and EFT (mm). 

In order to evaluate the degree of correlation between all variables, a Pearson’s correlation matrix was calculated. The association between SIRT1, adiponectin, leptin and each adiposity indicator (BMI, total FM %, trunk FM %, abdominal VAT, EFT, liver steatosis) was evaluated by using a linear regression model, taking into account the concomitant effect of age and gender (β, *p*-value). 

We evaluated the predictive power of SIRT1, adiponectin and leptin for each fat storage indicator by comparing the goodness of fit of each regression model using the coefficient of determination R squared (R^2^). This describes the proportion of the variance in the dependent variable (adiposity indicator) that is predictable from the independent variable (SIRT1, adiponectin, leptin). 

Data were analyzed with the use of STATA software, version 13 (STATACorp., College Station, TX, USA).

## 3. Results

Patients affected by AN were 1 male and 26 females, aged 18–51 years, with a BMI between 10.63 and 18.4 kg/m²; the NW subjects were 7 males and 19 females, aged 18–54 years, with a BMI between 19.4 and 24.4 kg/m²; and patients affected by obesity were 3 males and 24 females, aged 18–57 years, with a BMI between 30 and 49.6 kg/m². 

Table 1 shows the main characteristics of the patients stratified according to their BMI. Briefly, the mean BMI was 15.9 ± 2.2 kg/m^2^, 22.1 ± 1.6 kg/m^2^ and 38.3 ± 4.8 kg/m^2^ in AN, NW and patients with obesity, respectively. FM stores, indicative of mixed (subcutaneous plus visceral) fat depots (total FM %, trunk FM %) or visceral fat depots (abdominal VAT, EFT and liver steatosis), were all statistically different among the groups, with a trend to increase in parallel to the increase in BMI. The average total FM % was 17.41 ± 6.9%, 27.04 ± 6.2% and 41.04 ± 4.1% in patients with AN, NW and obesity, respectively, with a corresponding trunk FM % of 12.3 ± 6.1%, 22.7 ± 6.7% and 39.1 ± 4.2%. 

Mild-degree liver steatosis was present in 24 out of 27 patients with AN, in 5 out of 26 NW and in 19 out of 27 patients suffering from obesity. The remaining eight patients with obesity showed a fatty liver disease of moderate/severe degree. 

SIRT1, adiponectin and leptin levels were statistically different among the groups (*p* < 0.005). SIRT1 decreased significantly with the increase of FM (AN 3.39 ± 2.2 ng/mL; NW 2.47 ± 0.9 ng/mL; obesity 1.60 ± 1.8 ng/mL) in parallel with adiponectin (AN 6.39 ± 1.7 µg/mL; NW 4.82 ± 1.3 µg/mL; obesity 4.3 ± 1.7 µg/mL); leptin concentration increased with the expansion of FM (AN 5.63 ± 7.6 ng/mL; NW 6.13 ± 6.0 ng/mL; obese 23.75 ± 17.7).

**Figure 2:**
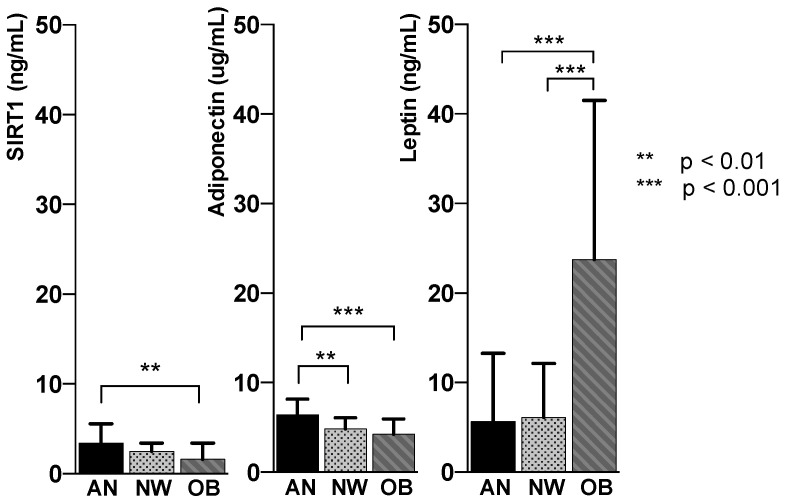
Representation of circulating SIRT1, adiponectin and leptin mean values in anorexia nervosa (AN, black column), normal weight (NW, gray column with dots) and obesity (OB, gray column with stripes).

An inverse relationship between SIRT1 and adiponectin and FM on one side, and the direct relationship between leptin and FM on the other, was observed in all groups. Thus, given the lowest amount of FM, the patients with AN showed the highest plasma levels of SIRT1 and adiponectin and the lowest concentration of leptin, while the opposite was found in the group of patients with obesity (Figure 2). 

Table 2 shows the association between each FM indicator and plasma SIRT1, adiponectin and leptin adjusted for the concomitant effect of age and gender. A significant inverse relationship was found among BMI (β = −0.066, *p* = 0.002), total FM % (β = −0.060, *p* = 0.001), trunk FM % (β = −0.048, *p* = 0.004), EFT (β = −0.379, *p* = 0.002) and SIRT1. We observed a trend of significant correlation of SIRT1 levels with abdominal VAT (β = −0.001, *p* = 0.067); the small sample size could have partially accounted for the different results found compared to other adiposity markers. No association was seen between liver steatosis and SIRT1 or adiponectin. Adiponectin was significantly associated to the other FM parameters. Finally, plasma leptin was positively associated with each variable of adiposity (*p* < 0.001). 

Regardless of the group, crude weight was never significantly associated with SIRT1, leptin and adiponectin, suggesting that the amount and distribution of the adipose tissue, more than weight per se, address these markers.

Table 3 shows the value of the coefficient of determination R^2^ used to determine the goodness of fit of each regression model in order to evaluate the strength of SIRT1 in predicting FM compared to adiponectin and leptin. The analysis was adjusted for age and gender.

Overall, SIRT1 showed a good ability in predicting FM distribution. The highest SIRT1 R^2^ value was observed for abdominal VAT (R^2^ = 0.61 in NW) and EFT (R^2^ = 0.73 in obesity), both visceral fat depots, while the lowest SIRT1 R^2^ value was observed for total FM % (R^2^ = 0.13 in AN) and trunk FM % (R^2^ = 0.07 in AN, R^2^ = 0.07 in NW, R^2^ = 0.12 in obesity), both mixed fat depots. Irrespective of the group of patients, SIRT1, adiponectin and leptin showed a substantially overlapping strength of prediction for EFT and liver steatosis. 

SIRT1 had a high predictive power mostly in the group of patients with obesity, where we observed no substantial differences in the predictive power of the three plasma variables. On the contrary, in conditions of severe fat loss like in AN, SIRT1 and adiponectin were not good predictors of FM, while leptin had the best predictive performance. 

**Figure 3:**
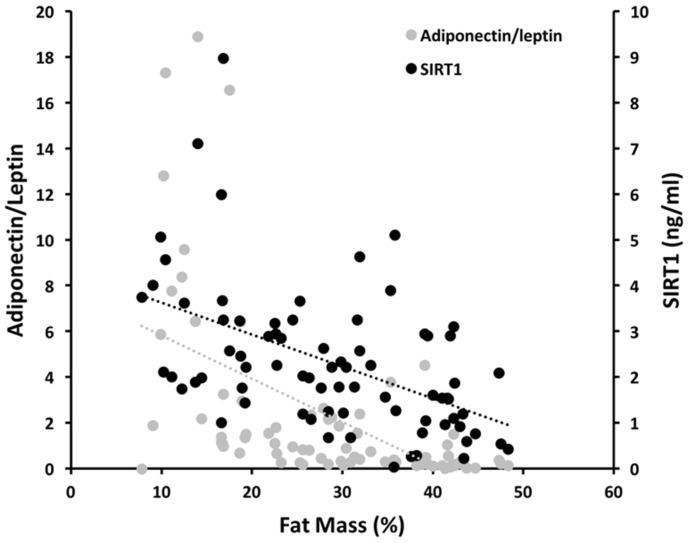
SIRT1 plasma concentration and adiponectin/leptin ratio in relation to the adipose tissue expansion. Grey dots represent the adiponectin/leptin ratio; black dots are the SIRT1 single values. Linear regression between SIRT1 and fat mass % (black broken line); linear regression between adiponectin/leptin ratio and fat mass % (grey broken line).

Figure 3 shows the evolution of SIRT1 plasma levels compared with the adiponectin/leptin ratio as the adipose tissue increases. This analysis confirms a negative correlation between both plasma concentrations of SIRT1 and the adiponectin/leptin ratio with FM % (*r* = −0.513, *p* = <0.0001; and *r* = −0.587, *p* = <0.0001; respectively). 

## 4. Discussion

The progress of recent decades in establishing adipose tissue as being essential in the regulation of energy homeostasis has been enormous. We herein evaluated the circulating concentration of SIRT1 in relation to the adipokines leptin and adiponectin under conditions of different amounts of adipose tissue. Our results show that bloodstream SIRT1 progressively decreases with the increase of the adipose tissue, following a pattern that is coherent with that of adiponectin and leptin. The positive relationships of SIRT1 with adiponectin and the negative relationship with leptin were congruent with both fat amount and distribution in all the categories of fat abundance tested, from AN through NW to obesity. These findings make the peripheral evaluation of SIRT1 a plausible clinical/biochemical parameter associated with adiposity.

Overnutrition-induced repletion of fat stores in obesity, as well as starvation-induced depletion of fat stores in AN, are both associated to the dysfunction of adipose tissue, and are accompanied by alterations in tissue and plasma adipokines and other adipocyte-derived factors [37,38,39,40]. Adipokines regulate several homeostatic processes and respond concurrently to various stimuli interacting with nutrients, hormones and metabolic sensors. Adiponectin and leptin are both circulating adipose tissue markers and central and peripheral mediators of energy homeostasis. Adiponectin negatively correlates with FM accumulation and modulates a number of metabolic processes, including inflammation, glucose regulation and fatty acid oxidation, resulting in an improvement in whole-body metabolism [41]. Leptin acts centrally as an anorexigenic hormone, promoting satiety and energy expenditure. Its concentration increases together with the expansion of FM and, conversely, leptin levels fall during starvation contributing to endocrine dysfunctions and reduced energy expenditure in the case of AN [5,42].

Both adiponectin and leptin have strict relationships with SIRT1. Adiponectin upregulates SIRT1 in vitro and in vivo via AMPK signaling [43], and SIRT1 acts as a positive regulatory feedback loop for adiponectin. SIRT1 and adiponectin together operate to adjust the gluco-lipidic profile, to mediate the white adipose tissue browning [44] and, most importantly, to antagonize insulin resistance, fatty liver diseases and inflammatory responses.

Leptin activates central SIRT1, which is essential for leptin-induced anorexic effects in the hypothalamic POMC neurons [45]. Leptin receptor mutant db/db mice [46] as well as leptin-deficient ob/ob mice [47] show a lack of SIRT1 activation in the hypothalamus in response to CR. According to previous studies, we found that the depletion of fat stores in AN is accompanied by a decrease of circulating leptin and a concomitant increase in adiponectin [8,39]. These evidences, coupled with our data on SIRT1, confirm that CR is a powerful stimulus for plasma SIRT1, and suggest that adiponectin, leptin and SIRT1 move peripherally in a coordinated manner, keeping metabolic flexibility against the metabolic damages in AN [6,39,48]. Moreover, in the case of deficiency of energy supplies, they point to restore the energy reserves required for survival.

We found a parallel but opposite behavior of SIRT1, leptin and adiponectin in patients with obesity. In view of the beneficial effects of both adiponectin and SIRT1 on metabolic health, their reduction in concentration in obesity could be either a mirror or a contributing factor to the deleterious effects of excess adipose tissue. The evaluation of peripheral SIRT1 across the broad spectrum of adiposity showed that plasma SIRT1 moves analogously to the expression pattern of SIRT1 mRNA seen within the adipose tissue [14]. Although our data do not allow the conclusion to be made that circulating SIRT1 is a mirror of tissue concentration, the low values of SIRT1 found in the face of high leptin levels when excess adipose tissue occurs could be a contributing factor to the resistance to leptin response constantly seen in patients with obesity. Interestingly, the modulation of hypothalamic SIRT1 has been implicated in the reduction of leptin resistance as a strategy for treating obesity [49,50], and the activation of SIRT1 by resveratrol improves peripheral and central leptin signaling and reverses leptin resistance caused by diet-induced obesity [51].

Recent data indicate that visceral fat expansion and ectopic fat distribution, more than the increased general adiposity phenotype, are determinant for cardiovascular and metabolic derangements and even fatal outcomes in obese patients [52,53,54]. We found that circulating SIRT1 correlates well with visceral fat and has a greater R^2^ value in predicting FM compared with leptin and adiponectin in patients with obesity. The inverse association between SIRT1 and visceral adiposity highlights that the reduction of SIRT1 might enhance the risk for the development of metabolic diseases. Notably, the reduction of SIRT1 promotes adipogenesis of the visceral adipose-derived stem cells (ASC), and patients with obesity exhibit significant lower SIRT1 mRNA expression in visceral ASC compared to subcutaneous ASC [17,55]. We recorded a high R^2^ coefficient of SIRT1 and adiponectin with the cardiac visceral EFT in the group of patients with obesity. These data confirm our previous observation of the negative correlation of SIRT1 with EFT [18], and are in line with the finding that nutrient restriction upregulates adiponectin in epicardial fat [8].

Moreover, we found liver steatosis in patients affected by obesity and, in a less severe form, in the majority of patients with AN. SIRT1 has important roles in maintaining normal liver function and its activation has beneficial effects against fatty liver disease [56]. Whether the higher levels of SIRT1 and adiponectin found in AN may be protective against more severe forms of liver steatosis deserves further investigation.

Obesity is characterized by increased and decreased levels of leptin and adiponectin, respectively. This translates in a reduction in the adiponectin/leptin ratio, which is indicative of a dysfunctional adipose tissue. There is a strong inverse correlation between this ratio and markers of low-grade chronic inflammation and metabolic syndrome-associated cardio-metabolic risk [57]. The parallel trend between plasma SIRT1 and adiponectin/leptin ratio that we recorded here suggests that SIRT1 could represent a clinically useful tool to identify subjects susceptible to cardio-metabolic diseases.

The relatively small sample size, mainly due to the difficulty in recruitment of patients with AN, together with the lack of concomitant evaluation of SIRT1, leptin and adiponectin tissue expression, may be potential limitations of our study. Further studies investigating these markers at a tissue level are needed to shed light on the underlying pathophysiology. However, SIRT1 plasma levels behave coherently with the expected values of adiponectin and leptin, established markers of adipose tissue amount and distribution, and altogether they are indicators of caloric intake and nutrient availability.

## 5. Conclusions

In conclusion, adipose tissue excess, as well as adipose tissue reduction, is characterized by significant modification in the concentration of circulating SIRT1. We propose that circulating SIRT1, which behaves coherently with FM and in synchrony with leptin and adiponectin, may represent a novel candidate to explore the entity of fat depots and metabolic health, aiding the evaluation of the risk of adverse outcomes related to fat mass, similarly to leptin and adiponectin.

Healthy mutual interaction between SIRT1, adiponectin and leptin is central in maintaining a stable metabolic homeostasis in different pathophysiological conditions. Having more affordable and predictable clinical markers is a starting point to limit the early onset of metabolic damage, and we suggest that the coordinated pattern of SIRT1 with leptin and adiponectin may have some pathophysiological relevance. Further investigation to verify whether SIRT1 may act as a potential target for the treatment of metabolic disorders or help monitoring the effect of new drugs to improve the energy metabolism is warranted.

## Figures and Tables

**Table 1:** Characteristics of the study population.

	Anorexia Nervosa	Normal Weight	Obesity	*p*-Value
*N.* subjects	27	26	27	-
Gender (male/female)	1/26	7/19	3/24	-
Age (years)	26.9 ± 12.2	29.5 ± 8.1	36.7 ± 12.4	<0.005
Weight (kg)	42.8 ± 8.7	64.8 ± 8.6	103 ± 16.25	<0.005
BMI (kg/m^2^)	15.9 ± 2.2	22.1 ± 1.6	38.3 ± 4.8	<0.005
Plasma markers of adiposity				
SIRT1 (ng/mL)	3.39 ± 2.2	2.47 ± 0.9	1.60 ± 1.8	<0.005
Adiponectin (µg/mL)	6.39 ± 1.7	4.82 ± 1.3	4.3 ± 1.7	<0.005
Leptin (ng/mL)	5.63 ± 7.6	6.13 ± 6.0	23.75 ± 17.7	<0.005
Districts of adiposity				
Total FM (%)	17.41 ± 6.9	27.04 ± 6.2	41.04 ± 4.1	<0.005
Trunk FM (%)	12.3 ± 6.1	22.7 ± 6.7	39.1 ± 4.2	<0.005
Abdominal VAT (g)	83.2 ± 62.6	223.8 ± 102.6	575.6 ± 226	<0.005
EFT (mm)	4.0 ± 0.7	6.5 ± 0.7	8.2 ± 0.7	<0.005
Liver Steatosis (degrees) *				
Absent	3 (11%)	21 (81%)	0 (0%)	<0.005
Mild	24 (89%)	5 (19%)	19 (70%)	<0.005
Moderate/severe	0 (0%)	0 (0%)	8 (30%)	<0.005

SIRT1, sirtuin 1; BMI, body mass index; FM, fat mass; VAT, visceral adipose tissue; EFT, epicardial fat thickness. * The severity of liver adiposity was based according to the brightness of the liver estimated as a numerical value: 0 = absent; 1 = mild lipid accumulation; 2 = moderate/severe lipid accumulation. Values are expressed as means ± SD.

**Table 2:** Association between fat indicators and SIRT1, adiponectin and leptin adjusted for age and gender.

	SIRT1		Adiponectin		Leptin	
	β	*p*-Value	β	*p*-Value	β	*p*-Value
BMI (kg/m^2^)	−0.066	0.002	−0.085	<0.001	0.84	<0.001
Weight (kg)	−0.002	0.2	−0.003	0.068	0.004	0.623
Total FM (%)	−0.06	0.001	−0.077	<0.001	0.672	<0.001
Trunk FM (%)	−0.048	0.004	−0.076	<0.001	0.62	<0.001
Abdominal VAT (g)	−0.001	0.067	−0.003	<0.001	0.032	<0.001
EFT (mm)	−0.379	0.002	−0.549	<0.001	4.328	<0.001
Liver steatosis (degrees)	−0.456	0.219	−0.027	0.942	9.576	<0.001

SIRT1, sirtuin 1; BMI, body mass index; FM, fat mass; VAT, visceral adipose tissue mass; EFT, epicardial fat thickness.

**Table 3:** Measures of performance of SIRT1, adiponectin and leptin to predict FM distribution by linear regression squared coefficient (R^2^).

	Anorexia Nervosa	Normal Weight	Obesity
Total FM %			
SIRT1	0.13	0.27	0.36
Adiponectin	0.08	0.42	0.36
Leptin	0.56	0.29	0.36
Trunk FM %			
SIRT1	0.07	0.07	0.12
Adiponectin	0.05	0.35	0.12
Leptin	0.55	0.06	0.13
Abdominal VAT			
SIRT1	0.17	0.61	0.21
Adiponectin	0.17	0.61	0.32
Leptin	0.32	0.53	0.26
EFT			
SIRT1	0.12	0.42	0.73
Adiponectin	0.13	0.47	0.72
Leptin	0.15	0.42	0.73
Liver steatosis			
SIRT1	0.16	0.16	0.32
Adiponectin	0.12	0.03	0.36
Leptin	0.11	0.04	0.37

SIRT1, sirtuin 1; FM, fat mass; VAT, visceral adipose tissue; EFT, epicardial fat thickness.
